# Inflammation, oxidative stress and purinergic signaling in pituitary neuroendocrine tumors (PitNETs)

**DOI:** 10.1007/s11302-026-10174-2

**Published:** 2026-07-07

**Authors:** Yenidis Teilor Scheibel, Symon Martins, André Paulo Turcatel, Francini Franscescon, Débora Tavares de Resende e Silva

**Affiliations:** 1 Medicine Department, Federal University of Fronteira Sul (UFFS), Chapecó, Santa Catarina Brazil; 2 Graduate Program in Biomedical Sciences, Federal University of Fronteira Sul (UFFS), Rodovia SC 484 - Km 02, Fronteira Sul, Chapecó, Santa Catarina CEP 89815-899 Brazil

**Keywords:** Pituitary neuroendocrine tumors, Purines, Oxidative stress, Tumor microenvironment, Metabolic reprogramming

## Abstract

Pituitary neuroendocrine tumors (PitNETs) constitute a heterogeneous group of intracranial neoplasms with variable biological behavior, whose aggressiveness cannot be explained solely by the intrinsic characteristics of tumor cells. Growing evidence demonstrates that chronic inflammation of the tumor microenvironment (TME) plays a central role in the progression, invasiveness, therapeutic resistance, and recurrence of these tumors. In this context, the purinergic system emerges as a fundamental regulatory axis of the local inflammatory response, integrating signals derived from cellular stress, hypoxia, and metabolic reprogramming. The extracellular release of ATP under conditions of oxidative stress, tissue damage, and cell death serves as a pro-inflammatory signal by activating P2 purinergic receptors. On the other hand, its conversion to adenosine by the ectonucleotidases CD39 and CD73 promotes an immunosuppressive environment, mainly through P1 receptors, such as A2A and A2B. This dynamic balance modulates the infiltration and polarization of immune cells, the production of inflammatory cytokines, including IL-6, IL-8, and TNF-α, the activation of transcriptional pathways such as NF-κB, STAT3, and HIF-1α, and extracellular matrix remodeling and angiogenesis. This study aims to review the role of inflammation in the tumor microenvironment of PitNETs, with emphasis on purinergic signaling as an integrating link between oxidative stress, immune dysfunction, and metabolic reprogramming. Although not abundantly explored, the purinergic pathway in PitNETs shows promising potential: it interconnects different elements of the TME, contributes to tumor mechanisms, and is a potential target for immunomodulatory and anti-inflammatory therapies in pituitary tumors.

## Highlights

• PitNETs are tumors significantly influenced by chronic inflammatory microenvironments.

• IL-6/STAT3 signaling emerges as a prominent inflammatory hub in PitNET progression.

• Purinergic signaling links inflammation, oxidative stress and metabolic reprogramming.

• This integrated model provides new insights into invasiveness, immune evasion, and therapy resistance.

• New inflammatory and immunometabolic targets are proposed for aggressive PitNET.

## Introduction

Pituitary neuroendocrine tumors (PitNETs) represent approximately 10 to 20% of intracranial neoplasms and exhibit a wide spectrum of clinical behavior, ranging from indolent lesions to locally invasive, recurrent tumors resistant to conventional therapies [1]. Although most present with benign histology, a significant proportion manifest aggressive growth, approximately 7 to 35% of cases [2], with persistent hormonal hypersecretion and invasion of adjacent structures, resulting in high morbidity [1, 3, 4]. These aspects indicate that the tumor biology of PitNETs cannot be understood solely in terms of classical proliferative mechanisms. In recent years, the tumor microenvironment (TME) has been recognized as a central determinant of tumor progression. In PitNETs, the TME constitutes a dynamic ecosystem composed of tumor, stromal, and immune cells, sustained by a complex network of cytokines, chemokines, and inflammatory metabolites. In this context, chronic inflammation emerges as a structuring process of tumor progression, modulating angiogenesis, extracellular matrix remodeling, immune evasion [5], and metabolic adaptation [6, 7].

Inflammatory responses in the PitNET tumor microenvironment may be strongly affected by purinergic signaling. Tumor cells subjected to oxidative stress, hypoxia, or death release extracellular adenosine triphosphate (ATP), which acts as a pro-inflammatory danger signal [8]. This activates P2 family receptors and triggers inflammatory processes, such as calcium influx, NLRP3 inflammasome activation, and increased cytokine production, including interleukin 1β (IL-1β) and tumor necrosis factor α (TNF-α) [9]. In contrast, ATP is rapidly degraded to adenosine by ectonucleotidases CD39 and CD73, promoting an immunosuppressive environment via P1 receptors (notably A2A and A2B). This suppresses cytotoxic responses and expands regulatory cell populations [10]. Oxidative stress is also a potentially central mechanism to tumor inflammation in PitNETs. High levels of reactive oxygen species (ROS) stem mainly from mitochondrial dysfunction and altered tumor metabolism. ROS cause not only cellular damage, but also regulate inflammatory pathways, including hypoxia-inducible factor 1α (HIF-1α) [6, 7], signal transducer and activator of transcription 3 (STAT3) [11, 12], and nuclear factor kappa B (NF-κB) [13].

These pathways promote the sustained expression of pro-inflammatory cytokines and angiogenic factors, establishing a feedback loop between inflammation, oxidative stress, and tumor progression. Thus, chronic inflammation, purinergic signaling, and oxidative stress constitute interdependent mechanisms that can underpin the aggressive phenotype of PitNETs [14]. Understanding the integration of these pathways is fundamental to developing new diagnostic and therapeutic strategies, especially those aimed at modulating the tumor microenvironment and the inflammatory response associated with tumor progression. This study proposes an integrated and innovative view of PitNET biology, demonstrating that its progression is sustained by interconnected circuits between chronic inflammation, IL-6/STAT3 signaling, the purinergic system, oxidative stress, and metabolic reprogramming [11, 13]. This review seeks to bring these processes together into a unified model, revealing biological convergence that favors invasiveness, immunosuppression, and therapeutic resistance. This integration highlights inflammatory and immunomodulatory targets that remain underexplored in PitNETs, thereby expanding the field’s translational potential.

### Pituitary neuroendocrine tumors (PitNETs)

Pituitary neuroendocrine tumors (PitNETs) are benign tumors that form in the anterior lobe of the pituitary gland and are the second most common primary intracranial tumor [15], accounting for approximately 10–20% of intracranial tumors [1]. In general, PitNETs can be classified using various criteria, including hormone production, cell of origin, anatomical-radiological characteristics, immunohistochemistry, and molecular patterns [16].

The change in nomenclature from pituitary adenoma to pituitary neuroendocrine tumor (PitNET) was introduced because the term “adenoma” conveys the notion of an invariably benign lesion, which does not accurately reflect the clinical and biological reality of these neoplasms. Many of these tumors exhibit invasive behavior, frequent recurrence, and therapeutic resistance, leading to significant morbidity in patients even in the absence of metastases. The previous classification, based solely on the presence of metastatic spread or on poorly reproducible criteria such as Ki-67 index and p53 assessment, failed to reliably predict tumor progression and prognosis. In contrast, the new terminology PitNET is consistent with that applied to other neuroendocrine tumors, acknowledges the heterogeneous spectrum of aggressiveness and patient impact, and allows these lesions to be considered within the oncological framework, thereby facilitating more accurate registry inclusion and access to appropriate therapies [17].

The increasingly frequent use of neuroimaging techniques has facilitated the unexpected identification of pituitary tumors, often during the clinical evaluation of nonspecific symptoms such as headache, epileptic seizures, syncope, or cranial trauma [18]. Consequently, the diagnosis of incidental PitNETs, also referred to as incidentalomas, has become more common [19]. Tumors that present with symptomatic complaints and are subsequently confirmed diagnostically through imaging exams, hormonal tests, and surgical and pathological confirmation are considered clinically relevant [18]. Among PitNETs, approximately ⅔ are functional, that is, they secrete hormones at clinically relevant levels [16].

Functional PitNETs cause abnormal hormonal hypersecretion, which can result in significant endocrine symptoms such as acromegaly, Cushing’s disease, galactorrhea, or hypogonadism [19], which is associated with high morbidity and mortality rates, even if they do not qualify as metastatic or malignant tumors [15]. In addition, they can be considered aggressive tumors, in 7 to 35% of cases, with high associations of morbidity and mortality [2] when high serum hormone levels, invasion of neighboring anatomical structures, high proliferative activity, rapid growth, poor response to conventional therapies, and/or recurrence regardless of the treatments performed are observed [20].

Regarding size, PitNETs smaller than 10 mm are classified as microadenomas, those larger than 10 mm as macroadenomas, and giant PitNETs as those larger than 40 mm [15]. Most cases are asymptomatic pituitary microincidentalomas without tumor growth. On the other hand, macroincidentalomas are more likely to grow and cause more severe symptoms, thus requiring active treatment [18]. Giant adenomas are generally benign, slow-growing, and non-functioning, with a higher prevalence in men. They can cause compression of surrounding brain structures, with visual and visual field impairments being the most common alterations, followed by headache, hypopituitarism, and hormonal hypersecretion [21]. Histological staging for PitNETs is based on tumor size, invasion into adjacent structures, and metastatic potential, which distinguishes typical PitNETs from pituitary carcinomas [15].

Regarding invasive capacity, PitNETs generally remain restricted to the sella turcica [16]. However, in invasive cases, the affected structures can be meningeal, such as the basal dura mater, the medial wall of the cavernous sinus, and the diaphragma sellae; bony structures, such as the sellar floor and the sphenoid bone; as well as the respiratory mucosa of the sphenoid sinus and, in some cases, brain tissue. PitNET invasion into the cavernous sinus and/or sphenoid sinus occurs in approximately 40% of surgical resections. Cavernous sinus invasion hinders surgical resection, increasing residual tumor size, and impairs treatment for hormonal secretion remission, with these cases being more prone to recurrence [15].

Furthermore, damage to surrounding pituitary structures and systemic hormonal changes also affect patients with PitNETs. In a recent study, [21] demonstrated that patients undergoing PitNET resection presented, compared to healthy controls, significantly elevated serum levels of extracellular ATP, with reduced capacity for ATP hydrolysis by peripheral blood mononuclear cells, in addition to alterations in cytokine levels, with increased interleukins IL-6, IL-10, and TNF-α and reduced IL-27. This represents a chronic state of systemic inflammation [21].

### The purinergic system in tumors

The purinergic system is a cellular communication pathway mediated by nucleotides, such as ATP, and nucleosides, such as adenosine and adenine, which act as extracellular signaling molecules. Under physiological conditions, their concentrations are low, but in the tumor microenvironment (TME), ATP is abundantly released by stressed or dying cells, acting as a “danger” signal [22]. These molecules activate purinergic receptors, regulating crucial biological processes such as cell proliferation, inflammation, and immune response [22, 23].

Purinergic signaling occurs through three receptor families: P0, activated by adenine; P1, activated by adenosine; and P2, activated by ATP, which regulate processes such as proliferation, cell death, inflammation, and immune response [22, 24]. Members of the P0 family are G protein-coupled receptors [25], as is the P1 family, which includes A1 and A3 receptors associated with Gi proteins, and A2A and A2B receptors associated with Gs proteins [26]. On the other hand, the P2 family, in addition to the G protein-coupled P2Y receptors (P2Y1, P2Y2, P2Y4, P2Y6, P2Y11 to P2Y14), also includes the P2X ion channels (P2X1) [23]. Virtually all cells express P1 and P2 receptors and are capable of transmembrane release of nucleotides into the extracellular environment [27, 28].

Ectonucleotidases are enzymes that hydrolyze extracellular nucleotides, primarily ATP, adenosine diphosphate (ADP), adenosine monophosphate (AMP), adenosine, and NAD+, generating metabolites relevant to the immune and inflammatory response. They are expressed by immune system cells, mainly on the cell surface, and are classified into four main families: ectonucleoside triphosphate diphosphohydrolases (NTPDases1/CD39), nicotinamide adenine dinucleotide glycohydrolase (NAD glycohydrolase/CD38), ecto-5′-nucleotidase (CD73), and ectonucleotide pyrophosphatases/phosphodiesterases (NPPs). Extracellular ATP is sequentially hydrolyzed to ADP and AMP by CD39 or directly hydrolyzed to AMP by NPPs. AMP can also be generated from NAD+ through the sequential activity of CD38 and NPP1 and is catabolized into adenosine by CD73. This sequential degradation mechanism sequesters extracellular ATP and generates intermediates with distinct signaling properties [10, 29]. In TME, the CD39/CD73 cascade primarily hydrolyzes ATP into adenosine and is essential for the interaction between tumor and immune cells [10].

## Purinergic system and inflammation modulation in PitNETs

Purinergic signaling is present in the pituitary gland and plays a significant role in its physiology, with both structural and secretory cells expressing distinct P1 and P2 receptors. In gonadotropic pituitary cells, particularly those that generate spontaneous action potentials, ATP modulates hormone release, intracellular Ca2+ oscillations, and action potential currents. These cells possess P2X2 heteromeric receptors, which modulate the frequency of action potentials and generate Ca2+ influx. Meanwhile, in lactotrophic cells, a signaling cascade occurs in which ATP activates P2X4 receptors, inducing depolarization and Ca2+ influx via voltage-dependent channels. ADP activates P2Y1, inducing Ca2+ mobilization from the endoplasmic reticulum and hyperpolarization via SK-type K+ channels. Adenosine activates A1 receptors coupled to Gi proteins, reducing adenylate cyclase activation and the generation of cyclic adenosine monophosphate (cAMP), and inhibiting Ca2+ channels and inducing K+ channels [30].

High concentrations of extracellular ATP and tissue oxidative stress contribute to the constitution of an immunomodulatory signaling network in TME. Depending on the receptor subtype activated, they may be associated with various immunosuppressive and immunostimulatory pathways. P1 receptors have a well-established immunosuppressive action, while P2 receptors have diverse functions, including the regulation of inflammatory processes and signaling in cell death or proliferation [24]. In several tumors, hypoxia, persistent inflammation, and cell death are frequently observed. This promotes the extracellular release of nucleotides, mainly ATP [8]. In a recent study [31], demonstrated that a membrane pore for ATP efflux, Pannexin 1 (PANX1), is expressed in PitNET tumor cells and elevated in invasive tumors. The authors also linked the upregulation of PANX1, induced in GH3 and MMQ cells and in PitNET cell lines in rats, to invasion and proliferation capacity, ATP efflux, and the induction of glycolytic activity [31].

In the extracellular space, ATP at high concentrations acts as a molecular danger signal, initiating and sustaining pro-inflammatory responses through purinergic receptor activation, but it can exert different effects depending on the receptors and the tumor context. Activation of P2X and P2Y receptors can trigger various effects, such as Ca2+ influx, activation of intracellular kinases, and induction of classic inflammatory pathways, including the NF-κB complex, the mitogen-activated protein kinase (MAPK) pathway, and the NLRP3 inflammasome [32, 33]. In PitNETs, these pathways are involved in various tumor mechanisms, suggesting a potential role for extracellular ATP beyond its acute inflammatory mediator function.

Among the receptors, P2X7 demonstrates a central role in different tumors. In tumor development, the P2X7 receptor is widely expressed in neoplastic and immune cells, interfering with metabolism and cell proliferation, and inducing cell death via membrane macropore formation at high ATP concentrations. However, tumor cells can develop resistance mechanisms, including in response to stress generated by extracellular ATP [34, 35]. Furthermore, extracellular ATP can influence immune infiltration. Activation of P2Y receptors modulates leukocyte recruitment towards an immunosuppressive profile, composed of tumor macrophages, regulatory T cells (Tregs), and myeloid-derived suppressor cells (MDSCs). Conversely, P2X7 activation in Tregs has a cytotoxic effect, and ATP stimulation of dendritic cells potentiates the lymphocytic response, favoring the antitumor response [36].

In the study by [31], the authors found that P2X4 and P2X7 are expressed in PitNETs, with P2X7 involved in the invasiveness of GH3 cells. From this, they demonstrated that, in cells with high levels of PANX1, extracellular ATP induced calcium influx via P2X7, which triggered an increase in the expression of matrix metalloproteinases (MMPs), such as MMP2 and MMP9, in addition to cytoskeletal remodeling, without altering actin expression, resulting in greater tumor invasiveness [31]. In PitNETs, the presence of MMP2 and MMP9, type 4 collagenases, has been associated with cavernous sinus invasion, with MMP9 also associated with higher vascular density, worse prognosis, and recurrence, and possessing carcinogenic potential and interacting with pathways such as p38 and NF-κB [5].

In PitNETs, tumor progression is associated with the dysregulation of multiple intracellular signaling pathways, especially in aggressive tumors [13]. Among these, the MAPK cascade, particularly the ERK/MAPK axis, plays a central role in tumor proliferation and is strongly modulated by purinergic signaling. Extracellular ATP activates its metabotropic receptors, promoting MAPK activation, as demonstrated by P2Y2 receptor stimulation, which induces robust p38 MAPK activation and directly connects the purinergic signal to inflammatory responses [37]. ERK hyperactivation is frequent in recurrent prolactinomas and corticotropinomas, often driven by somatic mutations or epidermal growth factor receptor (EGFR) signaling, making this pathway a relevant therapeutic target [38, 39]. In non-functioning gonadotropic PitNETs (NFPAs), sustained ERK activation is amplified by purinergic signaling via P2X2 receptors, which promote Ca^2^⁺ influx and kinase-dependent activation, exacerbating the proliferative response in ATP-rich microenvironments [38, 40].

**Fig. 1:**
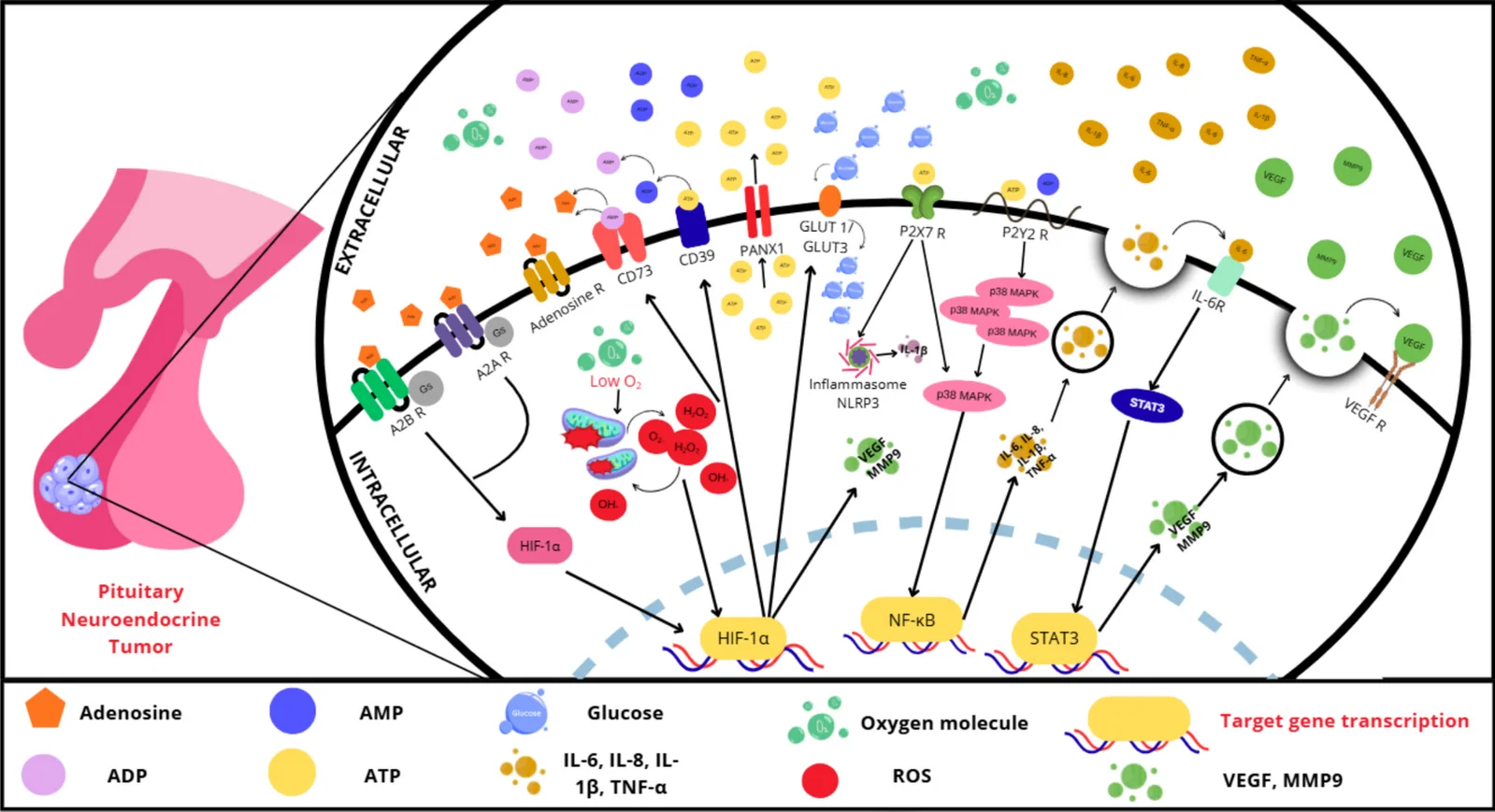
Purinergic signaling and inflammatory pathways in hypoxic pituitary tumor cells. The diagram represents a pituitary tumor cell under hypoxia, oxidative stress, inflammatory action, and purinergic signaling, highlighting ATP release through the PANX1 channel and its sequential degradation by CD39 and CD73 into ADP, AMP, and adenosine (ADO). The accumulation of ADO activates A2A and A2B receptors, stimulating the HIF-1α pathway, thereby inducing the expression of ectonucleotidases, GLUT1/GLUT3 transporters for glucose uptake, as well as VEGF and MMP9. Hypoxia-induced mitochondrial dysfunction enhances reactive oxygen species (ROS) production, reinforcing HIF-1α activation and contributing to the invasive phenotype. Extracellular ATP also activates P2 receptors, such as P2X7, which promotes the formation of the NLRP3 inflammasome and the release of IL-1β, in addition to stimulating p38 MAPK. P2Y2 activation also converges on p38 MAPK, which, together with NF-κB, induces the production of pro-inflammatory cytokines (IL-1β, IL-6, IL-8, and TNF-α). These cytokines exert autocrine effects on membrane receptors, activating the STAT3 pathway, which in turn increases the transcription of VEGF and MMP9, enhancing angiogenesis, matrix remodeling, and tumor progression

However, in addition to its role as a signaling molecule, extracellular ATP is hydrolyzed in the purinergic cascade, coordinated by the membrane enzymes CD39 and CD73 (Fig. 1), to generate adenosine. In their review, [10] highlight the role of CD39 in accelerating tumor growth and metastasis, with CD73 playing a crucial role by converting AMP into adenosine (ADO) [10].

However, in GH4 tumor cells, a PitNET cell line, adenosine has been shown to modulate L-type calcium channel activity [41]. ADO exerts immunosuppressive and anti-inflammatory effects, acting mainly on A2A and A2B receptors in immune cells [10], which are more efficient than mechanisms dependent on cell-to-cell contact by live Treg cells [39]. This superior efficiency is explained by the fact that apoptotic Tregs, as demonstrated by [42], release large amounts of ATP into the extracellular environment via Pannexin 1, which is rapidly converted into ADO by the action of overexpressed CD39 and CD73 on the membrane. In PitNETs, this suggests a paradoxical mechanism where tumor cell death, instead of limiting progression, might reinforce immunosuppression through this metabolic amplification. When it accumulates in the TME, adenosinergic signaling is able to suppress the cytotoxic response of T cells and natural killer (NK) cells, and can promote the stability and function of Tregs, which co-express CD39 and CD73, creating a feedback loop that amplifies ADO production and immunosuppression [10].

Thus, extracellular ATP accumulation, the CD39/CD73 axis, and the conversion of ATP to ADO represent an active mechanism of TME. In general, the purinergic system acts as a regulator of local chronic inflammation, controlling the dynamic balance between ATP-mediated pro-inflammatory signals and adenosine-induced immunosuppressive responses. Dysregulation of this system contributes, in complex ways, to the definition of various tumor characteristics, positioning purinergic signaling as an important integrator of inflammation, oxidative stress, tumor metabolism, and immune evasion [34]. The purinergic system, therefore, acts as a bridge between the metabolic state of the tumor and its immune landscape. Rather than being a mere consequence of cellular stress, the ATP/Adenosine balance actively interferes with the infiltration and polarization of the immune cells described in the following section.

## The mechanisms of immune dysregulation

Beyond the purinergic signaling balance, the resulting immune landscape is further shaped by specific cellular infiltrates, leading to broader immune dysregulation. The immune infiltrate of PitNETs (TME) reflects a dysfunctional inflammatory state with an immunosuppressive profile, with M2 macrophages as the main infiltrating population. Lymphocyte content is rare and mainly comprises memory CD4+ T lymphocytes [5]. More recent studies have shown that lymphocyte infiltration occurs mainly in producing PitNETs, with CD4+ T cells, CD8+ T cells, and B lymphocytes as the main components [43]. The main lymphocyte alteration described in PitNETs is a reduction in the CD8/CD4 and CD8/FOXP3 ratios, indicating decreased antitumor cytotoxic activity, an alteration associated with invasion, proliferation, and treatment resistance [5]. Treg cells are uncommon and are generally elevated in aggressive, poor-prognosis PitNETs [4]. This predominance of M2 macrophages and the reduced CD8/CD4 ratio suggest that PitNETs employ a strategy of immune escape rather than active inflammation.

Recent high-resolution mapping using single-cell RNA sequencing and spatial transcriptomics has further refined this immune landscape. [44] identified a specific subpopulation of SPP1+ tumor-associated macrophages (TAMs) that are significantly enriched in invasive PitNETs. These cells do not merely exist in the TME, they actively facilitate tumor progression by colocalizing with cancer-associated fibroblasts (CAFs) and promoting invasion through the SPP1-ITGAV/ITGB1 signaling pathway. This discovery provides a spatial and molecular context to the inflammatory axis discussed here, suggesting that the interaction between purinergic signaling and specific myeloid niches, such as these SPP1+ TAMs, may be a primary driver of the aggressive phenotype across various PitNET subtypes [44].

Furthermore [43], associated invasiveness in somatotropinomas with infiltration of neutrophils, eosinophils, and mast cells, and in thyrotropinomas with infiltration of monocytes, CD4+ T lymphocytes, and NK cells [43]. The high M2/M1 ratio found in PitNETs, especially in NFPAs, is a known tumor characteristic of immunosuppression. Tumor cells tend to select M2 lineages, either by chemotaxis via increased CC motif chemokine ligand 2 (CCL2) and decreased granulocytic macrophage colony stimulating factor (GM-CSF), or by polarization to the M2 phenotype. One of the main chemokines found in PitNETs is CCL2, released by tumor cells, which has also been associated with the recruitment of neutrophils and CD8+ T lymphocytes, mainly in NFPAs, and with proliferation and angiogenesis [5]. In their study [45], demonstrated that M2 macrophages exhibit elevated CD39 and CD73 activity compared to resident and M1 macrophages, highlighting the role of the purinergic cascade in the immunomodulatory activity of M2 polarization, by accumulating ADO and favoring P1 receptors [45].

The TME of PitNETs is also marked by the release of a variety of cytokines. Among the main ones, IL-8, CCL2-4, and IL-6 stand out. All PitNET subtypes showed increased expression of IL-17 and IL-36. IL-36 is a cytokine inducer of macrophages, T lymphocytes, and neutrophils, and can work synergistically with IL-22 to induce IL-17A and TNF-α, with which it also acts to increase IL-6 and IL-8 [43]. IL-17 can influence TME, potentially associated with invasiveness and MMP-9 activity [5].

In TME, this diversity of factors forms a dynamic ecosystem of complex interactions, in which tumor, stromal, and immune cells, along with the extracellular matrix, establish a signaling network through cytokines and chemokines. In this way, tumor processes and characteristics are shaped, such as progression, local invasion, immune response, therapeutic resistance, and recurrence [5]. In PitNETs, there is no dominant regulatory axis that characterizes these interactions [45], and the functions of signaling and transcription pathways, even if well described, cannot be understood in isolation [5].

Vascular endothelial growth factor (VEGF) is a factor that represents this interaction, being expressed in a cytokine-dependent manner in the normal pituitary gland and in prolactinomas, somatotropinomas, and NFPAs. This factor has been associated with cavernous sinus invasion and hemorrhage in TME. In NFPAs, tumor fibroblasts, relevant for bone invasion, induce the production of vascular endothelial growth factor A (VEGF-A). The VEGF-R1 receptor is also increased in cavernous sinus NFPAs, with the RWD-containing sumoylation enhancer protein (RSUME) and HIF-1α being factors that promote VEGF-A expression in invasive PitNETs [4]. Thus, increased VEGF-A expression, regulated by RSUME and HIF-1α, may contribute to the invasive behavior of PitNETs, especially NFPAs, suggesting its relevance as a potential marker of tumor aggressiveness and a therapeutic target in PitNETs.

Tumor fibroblasts (TAFs) are classically associated with malignancy and poor prognosis, found in the capsules and fibrous matrices, mainly of thyrotropinomas [5], and in refractory PitNETs [4]. The presence of TAFs has been linked to the activity of the TGF-β1/SMAD3 pathway, which is also associated with fibrosis and the inhibition of proliferation and hormone production. Tumor growth factor beta (TGF-β) plays an important role in mediating the inhibitory effect of dopamine on lactotrophic cells through SMAD2, 3, and 4 transducers, and is relevant in dopamine-resistant prolactinomas (Ben-Shlomo, 2023). In addition to VEGF-A, TAFs influence the TME by producing other cytokines associated with invasiveness, such as CCL2 and IL-8, FGF-2 (basic fibroblast growth factor), and IL-6 [5]. The interaction between these stromal cells and inflammatory mediators, particularly IL-6, establishes a feedback loop that reinforces the aggressive phenotype.

Among the inflammatory mediators present in the TME of PitNETs, interleukin-6 is among the main ones. IL-6 is more abundant in invasive tumors, such as PitNETs with a mutation in the aryl hydrocarbon receptor interacting protein (AIP) gene, which increases phosphorylated STAT3 levels. It has also been linked to hormone production, proliferation, interaction with VEGF for angiogenesis, and extracellular matrix (ECM) remodeling through MMP signaling [5]. However, its effect is dual: paracrine signaling has been shown to promote cell growth, while autocrine signaling has been linked to senescence, restricting aggressive growth and malignancy [5, 8]. This interleukin is released not only by the immune infiltrate but also by tumor cells and follicle-stellate cells (FSCs). FSCs, one of the main stromal components in the TME of PitNETs, have A2B receptors [41], P2Y2 [46], and P2X2 [47], which are important for regulating the release of signaling molecules such as IL-6, TNF, and nitric oxide [41].

In PitNETs, sustained activation of the IL-6/STAT3 pathway has been associated with aggressive behavior, invasive potential, and alterations in the tumor secretory profile [5]. This sustained activation is directly correlated with tumor invasiveness, through the induction of MMP9 via the IL-6 receptor, and with hormonal hypersecretion [11]. In this context, the purinergic system has the potential to act as a key inflammatory trigger. Activation of the P2X7 receptor promotes the assembly of the NLRP3 inflammasome and the release of IL-1β, in addition to stimulating the conversion of TNF-α through the p38 MAPK pathway. Establishing a direct link between excess ATP in the TME and sustained chronic inflammation favors the progression of PitNETs [9]. The p38 MAPK pathway has a dual function: it can induce apoptosis or, when phosphorylated, promote invasiveness and immune escape by inducing MMP9 [38].

When phosphorylated, this pathway cooperates with the NF-κB complex, resulting in the release of pro-inflammatory cytokines such as TNF-α and IL-1β. This, in turn, amplifies IL-6. Thus, the important interaction between these pathways reinforces sustained activation of the IL-6/STAT3 axis and MMP9 induction, thereby contributing to tumor aggressiveness and cavernous sinus invasion.

Persistent inflammation also has direct implications for bone invasion, a characteristic of aggressive PitNETs. In addition to IL-6, TNF-α has also been associated with invasive tumors and immune escape [48]. TNF-α is associated with bone invasion via osteoclast differentiation, reduced hormone release, and increased VEGF and MMP9 [5]. The purinergic system plays an important role in bone invasion, with activation of P2X7 receptors essential for regulating osteoclast activation [49], while P2X5 activation contributes to osteolysis and destruction of the sellar floor, favoring tumor expansion in an inflammatory TME [4]. In this way, purinergic signaling has the potential to reinforce and support the development of an aggressive phenotype and invasion mechanisms by interfering with the profiles of other signaling molecules, affecting multiple cellular pathways, and modulating ECM [34]. Taken together, these data indicate that the TME of PitNETs is sustained by an integrated chronic inflammation, in which pathways such as IL-6/STAT3, immunosuppressive immune infiltrate, and ECM remodeling act in a coordinated manner to promote tumor mechanisms. This chronic inflammatory state does not exist in isolation; it is intimately linked to the metabolic reprogramming and oxidative stress that are described in the PitNET microenvironment.

## Oxidative stress, metabolic reprogramming, and its relationship with purinergic signaling in PitNETs

Oxidative stress represents a central component of tumor biology and chronic inflammation in PitNETs. Oxidative stress is defined as an imbalance in the production of reactive oxygen species (ROS) and their removal through antioxidant defenses. The production of ROS, for example, superoxide anion (O2-), hydrogen peroxide (H2O2), and hydroxyl radical (OH•), stems mainly from mitochondrial dysfunction, hypoxia, and tumor metabolic reprogramming, being widely recognized as an active modulator of inflammatory signaling, but it can also be a source of cellular damage [49]. Patients with PitNETs showed increased serum levels of ROS and carbonylated proteins [21]. On the other hand, elevated levels of ascorbic acid, total thiols (SH), and non-protein thiols (NPSH) were detected [21]. Despite the increase in thiol levels, a chronic inflammatory profile, elevated ROS levels, and alterations in purinergic signaling frequently occur in patients with PitNETs.

The IL-6/STAT3 axis is a key point of convergence between oxidative stress and tumor inflammation. ROS can directly activate the JAK2/STAT3 pathway or inhibit phosphatases that regulate it, thereby sustaining STAT3 activation. Once activated, STAT3 stimulates the expression of genes involved in proliferation, apoptosis resistance, and metabolic adaptation [50]. Metabolic reprogramming of tumor cells directly contributes to this inflammatory scenario. Pathways such as MAPK, PKC-ε, and mTOR converge to phosphorylate STAT3 at serine residues (pS-STAT3), a specific post-translational modification that directs the protein to the mitochondria. Once in the organelle, pS-STAT3 optimizes the electron transport chain and promotes aerobic glycolysis (Warburg effect), allowing the tumor to sustain high proliferation rates even under intense oxidative stress [11].

STAT3 signaling establishes a positive feedback loop with inflammatory factors in the microenvironment, such as NF-κB and PAI-1, and modulates autocrine IL-6 secretion, which, in turn, activates the JAK2/STAT3 pathway in adjacent cells [13]. This sustained activation is directly correlated with tumor invasiveness, through the induction of MMP9 via the IL-6 receptor, and with hormonal hypersecretion (GH, IGF-1, PRL, and ACTH), consolidating STAT3 as a key tumor metabolic factor for defining tumor characteristics. This transcription factor is recognized as important for altering gene expression through cytokine signaling, associated with the expression of genes related to cell survival, angiogenesis, metastasis, and proliferation, and the silencing of growth suppressors [11].

This metabolic environment favors the stabilization of HIF-1α, which acts synergistically with STAT3 to induce genes such as VEGF, MMP9, GLUT1, and GLUT3, involved in angiogenesis, glycolytic metabolism, and immune evasion [50, 51]. In PitNETs, these metabolic adaptations contribute to tumor survival under adverse conditions and to increased invasive potential. One of the main consequences of HIF-1 activation is the induction of the Warburg effect. This phenomenon is not just a passive adaptation, but a reprogramming orchestrated by the overexpression of glycolytic enzymes. This machinery enables extremely accelerated ATP turnover by rapidly converting pyruvate to lactate via lactate dehydrogenase (LDH) [52]. In the specific context of pituitary tumorigenesis, the study by [6] demonstrated a progressive, sustained increase in lactate production during tumor development. The authors suggest that this metabolic shift is fundamental to providing the rapid ATP supply needed for the initial proliferative “burst.” Consequently, this accelerated metabolism creates the gradient necessary for ATP efflux via purinergic channels and promotes TME acidification, a key factor in M2 macrophage polarization [4]. Crucially, this metabolic shift indicates that invasiveness in PitNETs is not merely a proliferative trait, but a survival strategy. By acidifying the microenvironment and sustaining high ATP turnover, the tumor creates a niche that is chemically hostile to immune surveillance but favorable to its own structural remodeling.

Purinergic signaling integrates into this redox-inflammatory-metabolic axis. Extracellular ATP release acts as a danger signal by activating P2 receptors, notably P2X7, as reviewed by [32]; this activation not only amplifies the production of classic pro-inflammatory cytokines, such as TNF and IL-1β, through NF-κB and the inflammasome, but also induces the release of immunomodulatory factors such as IL-10 and TGF-β. Furthermore, P2X7 is a potent inducer of the HIF-1α pathway, further consolidating the link between the ATP-rich microenvironment and the hypoxic response [32].

In parallel, the conversion of ATP to adenosine by the ectonucleotidases CD39 and CD73 is favored in hypoxic conditions, leading to activation of A2A and A2B receptors [53]. The conversion of this ATP to adenosine reflects a complex adaptation: although oxidative stress tends to inhibit the enzymatic activity of ectonucleotidases [54], due to high levels of ROS, concomitant hypoxia induces gene overexpression of CD39 and CD73 via HIF-1 [22, 28]. This mechanism ensures continuous adenosine production, which, by activating A2 receptors, reinforces the tumor’s immunosuppressive profile. Furthermore, tumor hypoxia and mitochondrial oxidative stress stabilize HIF-1α, while IL-6-mediated inflammatory signaling promotes sustained STAT3 activation, which cooperates functionally with HIF-1α to induce glycolytic genes and suppress p53, favoring cell survival and proliferation [6, 7].

Purinergic signaling reinforces this inflammatory-metabolic axis. In the tumor microenvironment, released ATP is converted to adenosine by ectonucleotidases CD39 and CD73, and activation of the A2A receptor stimulates the cAMP/PKA-CREB pathway, promoting sustained transcription of HIF-1α [14].

Thus, ROS, IL-6/STAT3, HIF-1, and purinergic signaling do not act as independent processes, but as interconnected components of a functional circuit that sustains chronic inflammation, metabolic adaptation, and tumor progression in PitNETs. Taken together, these data indicate that oxidative stress and metabolic reprogramming play active roles in maintaining the inflammatory tumor microenvironment in PitNETs. Disrupting this redox-inflammatory-metabolic axis, therefore, represents a promising strategy for developing therapeutic approaches to modulate the tumor microenvironment and the aggressiveness of these tumors.

### Redox balance and cellular adaptation

The TME of PitNETs is characterized by chronic oxidative stress, whose impact on tumor progression depends on the balance between ROS production, inflammatory activation, and cellular antioxidant capacity [7]. The main enzymatic source of ROS involves the NADPH oxidase (NOX) family, whose activation can be induced by purinergic signaling. Stimulation of the P2X7 receptor in immune cells promotes the assembly of the NOX complex and the generation of the superoxide anion, a process dependent on Ca^2^⁺ influx that can also amplify mitochondrial ROS production [54–56]. This axis is finely modulated by receptors such as P2X4, which negatively regulates P2X7-mediated ROS production [48, 57, 58].

The relevance of this mechanism to pituitary tumors is underscored by the association between purinergic signaling and NOX-mediated oxidative stress, which can drive tumor progression [7]. Initial oxidative damage, observed in animals, can induce genomic instability and cellular senescence [6]; however, the subsequent activation of defense pathways allows tumor cells to adapt and survive in this hostile environment, possibly in a state of “programmed senescence” that restricts uncontrolled growth but prevents complete tumor elimination [59–61]. Therefore, it is not only the absolute levels of ROS, but rather the dynamic balance and adaptive capacity of the tumor cell that dictate the progression of PitNETs.

## Conclusions and perspectives

Evidence accumulated over the last decade demonstrates that PitNETs should be understood as neoplasms sustained by a chronic inflammatory TME, in which multiple signaling pathways interact to promote tumor progression, local invasiveness, and therapeutic resistance. In this context, inflammation is not a secondary phenomenon but a structuring component of tumor biology, integrating oxidative stress, metabolic reprogramming, immune dysfunction, and purinergic signaling.

In this respect, strategies aimed at modulating tumor inflammation, such as blocking the IL-6/STAT3 pathway, inhibiting CD39 and CD73 or A2A/A2B adenosinergic receptors, and interfering with redox and metabolic mechanisms, emerge as promising approaches. In conclusion, an integrated understanding of chronic inflammation, purinergic signaling, and metabolic reprogramming enables improved understanding of PitNET characteristics and the identification of potential therapeutic targets in the TME. A deeper understanding of the main mechanisms and their clinical and therapeutic implications for these tumors could not only improve therapies but also identify biomarkers that predict treatment response and recurrence risk, thereby advancing their management.

Several studies indicate that PitNETs exhibit substantial biological and immunological heterogeneity, which may directly influence the relevance and therapeutic applicability of inflammatory and purinergic signaling pathways. Functioning and non-functioning PitNETs differ in hormone secretion profiles, proliferative potential, immune cell infiltration, and expression of inflammatory mediators, suggesting that these mechanisms may not contribute uniformly across all tumor subtypes. For instance, aggressive corticotroph and sparsely granulated somatotroph tumors have been associated with increased inflammatory signaling and higher invasive potential compared to less aggressive subtypes. Likewise, differences in immune checkpoint expression and cytokine profiles among PitNET subtypes may affect responsiveness to immunomodulatory or purinergic-targeted therapies. Therefore, considering the intrinsic heterogeneity of PitNETs is essential for the interpretation of current findings and for the future development of personalized therapeutic strategies.

## Data Availability

No datasets were generated or analysed during the current study.
